# Supplementary material for: Resolving the full spectrum of human genome variation using Linked-Reads
Source: Genome Res. 2019 Apr;29(4):635–45. doi: 10.1101/gr.234443.118 (PMC6442396; doi:10.1101/gr.234443.118)
Supplement: Supplemental Material [file supp_gr.234443.118_Supplemental_longranger-2.2.zip › longranger-2.2.tar.bz2!/lr2.2-public/lib/stan/cmdstan/stan/doc/api/html/uniform_8hpp_source.html]

Stan: src/stan/prob/distributions/univariate/continuous/uniform.hpp Source File


|  |  |
| --- | --- |
|  | Stan  2.5.0  probability, sampling & optimization |


- Main Page
- Related Pages
- Namespaces
- Classes
- Files

- File List
- File Members

All Classes Namespaces Files Functions Variables Typedefs Enumerations Enumerator Friends Macros Pages

- src
- stan
- prob
- distributions
- univariate
- continuous

uniform.hpp

Go to the documentation of this file.

1 #ifndef STAN\_\_PROB\_\_DISTRIBUTIONS\_\_UNIVARIATE\_\_CONTINUOUS\_\_UNIFORM\_HPP

2 #define STAN\_\_PROB\_\_DISTRIBUTIONS\_\_UNIVARIATE\_\_CONTINUOUS\_\_UNIFORM\_HPP

3

4 #include <boost/random/uniform\_real\_distribution.hpp>

5 #include <boost/random/variate\_generator.hpp>

6

7 #include <stan/agrad/partials\_vari.hpp>

8 #include <stan/math/error\_handling.hpp>

9 #include <stan/math/constants.hpp>

10 #include <stan/math/functions/value\_of.hpp>

11 #include <stan/meta/traits.hpp>

12 #include <stan/prob/constants.hpp>

13 #include <stan/prob/traits.hpp>

14

15 namespace stan {

16

17  namespace prob {

18

19  // CONTINUOUS, UNIVARIATE DENSITIES

41  template <bool propto,

42  typename T\_y, typename T\_low, typename T\_high>

43  typename return\_type<T\_y,T\_low,T\_high>::type

44  uniform\_log(const T\_y& y, const T\_low& alpha, const T\_high& beta) {

45  static const char\* function = "stan::prob::uniform\_log(%1%)";

46

47  using stan::math::check\_not\_nan;

48  using stan::math::check\_finite;

49  using stan::math::check\_greater;

50  using stan::math::value\_of;

51  using stan::math::check\_consistent\_sizes;

52

53  // check if any vectors are zero length

54  if (!(stan::length(y)

55  && stan::length(alpha)

56  && stan::length(beta)))

57  return 0.0;

58

59  // set up return value accumulator

60  double logp(0.0);

61  check\_not\_nan(function, y, "Random variable", &logp);

62  check\_finite(function, alpha, "Lower bound parameter", &logp);

63  check\_finite(function, beta, "Upper bound parameter", &logp);

64  check\_greater(function, beta, alpha, "Upper bound parameter", &logp);

65  check\_consistent\_sizes(function,

66  y,alpha,beta,

67  "Random variable","Lower bound parameter",

68  "Upper bound parameter",

69  &logp);

70

71  // check if no variables are involved and prop-to

72  if (!include\_summand<propto,T\_y,T\_low,T\_high>::value)

73  return 0.0;

74

75  VectorView<const T\_y> y\_vec(y);

76  VectorView<const T\_low> alpha\_vec(alpha);

77  VectorView<const T\_high> beta\_vec(beta);

78  size\_t N = max\_size(y, alpha, beta);

79

80  for (size\_t n = 0; n < N; n++) {

81  const double y\_dbl = value\_of(y\_vec[n]);

82  if (y\_dbl < value\_of(alpha\_vec[n])

83  || y\_dbl > value\_of(beta\_vec[n]))

84  return LOG\_ZERO;

85  }

86

87  DoubleVectorView<include\_summand<propto,T\_low,T\_high>::value,

88  is\_vector<T\_low>::value | is\_vector<T\_high>::value>

89  inv\_beta\_minus\_alpha(max\_size(alpha,beta));

90  for (size\_t i = 0; i < max\_size(alpha,beta); i++)

91  if (include\_summand<propto,T\_low,T\_high>::value)

92  inv\_beta\_minus\_alpha[i]

93  = 1.0 / (value\_of(beta\_vec[i]) - value\_of(alpha\_vec[i]));

94  DoubleVectorView<include\_summand<propto,T\_low,T\_high>::value,

95  is\_vector<T\_low>::value | is\_vector<T\_high>::value>

96  log\_beta\_minus\_alpha(max\_size(alpha,beta));

97  for (size\_t i = 0; i < max\_size(alpha,beta); i++)

98  if (include\_summand<propto,T\_low,T\_high>::value)

99  log\_beta\_minus\_alpha[i]

100  = log(value\_of(beta\_vec[i]) - value\_of(alpha\_vec[i]));

101

102  agrad::OperandsAndPartials<T\_y,T\_low,T\_high>

103  operands\_and\_partials(y,alpha,beta);

104  for (size\_t n = 0; n < N; n++) {

105  if (include\_summand<propto,T\_low,T\_high>::value)

106  logp -= log\_beta\_minus\_alpha[n];

107

108  if (!is\_constant\_struct<T\_low>::value)

109  operands\_and\_partials.d\_x2[n] += inv\_beta\_minus\_alpha[n];

110  if (!is\_constant\_struct<T\_high>::value)

111  operands\_and\_partials.d\_x3[n] -= inv\_beta\_minus\_alpha[n];

112  }

113  return operands\_and\_partials.to\_var(logp);

114  }

115

116  template <typename T\_y, typename T\_low, typename T\_high>

117  inline

118  typename return\_type<T\_y,T\_low,T\_high>::type

119  uniform\_log(const T\_y& y, const T\_low& alpha, const T\_high& beta) {

120  return uniform\_log<false>(y,alpha,beta);

121  }

122

123  template <typename T\_y, typename T\_low, typename T\_high>

124  typename return\_type<T\_y,T\_low,T\_high>::type

125  uniform\_cdf(const T\_y& y, const T\_low& alpha, const T\_high& beta) {

126  static const char\* function = "stan::prob::uniform\_cdf(%1%)";

127

128  using stan::math::check\_not\_nan;

129  using stan::math::check\_finite;

130  using stan::math::check\_greater;

131  using stan::math::value\_of;

132  using stan::math::check\_consistent\_sizes;

133

134  // check if any vectors are zero length

135  if (!(stan::length(y)

136  && stan::length(alpha)

137  && stan::length(beta)))

138  return 1.0;

139

140  // set up return value accumulator

141  double cdf(1.0);

142  check\_not\_nan(function, y, "Random variable", &cdf);

143  check\_finite(function, alpha, "Lower bound parameter", &cdf);

144  check\_finite(function, beta, "Upper bound parameter", &cdf);

145  check\_greater(function, beta, alpha, "Upper bound parameter", &cdf);

146  check\_consistent\_sizes(function,

147  y,alpha,beta,

148  "Random variable","Lower bound parameter",

149  "Upper bound parameter",

150  &cdf);

151

152  VectorView<const T\_y> y\_vec(y);

153  VectorView<const T\_low> alpha\_vec(alpha);

154  VectorView<const T\_high> beta\_vec(beta);

155  size\_t N = max\_size(y, alpha, beta);

156

157  for (size\_t n = 0; n < N; n++) {

158  const double y\_dbl = value\_of(y\_vec[n]);

159  if (y\_dbl < value\_of(alpha\_vec[n])

160  || y\_dbl > value\_of(beta\_vec[n]))

161  return 0.0;

162  }

163

164  agrad::OperandsAndPartials<T\_y,T\_low,T\_high>

165  operands\_and\_partials(y,alpha,beta);

166  for (size\_t n = 0; n < N; n++) {

167  const double y\_dbl = value\_of(y\_vec[n]);

168  const double alpha\_dbl = value\_of(alpha\_vec[n]);

169  const double beta\_dbl = value\_of(beta\_vec[n]);

170  const double b\_min\_a = beta\_dbl - alpha\_dbl;

171  const double cdf\_ = (y\_dbl - alpha\_dbl) / b\_min\_a;

172

173  //cdf

174  cdf \*= cdf\_;

175

176  //gradients

177  if (!is\_constant\_struct<T\_y>::value)

178  operands\_and\_partials.d\_x1[n] += 1.0 / b\_min\_a / cdf\_;

179  if (!is\_constant\_struct<T\_low>::value)

180  operands\_and\_partials.d\_x2[n] += (y\_dbl - beta\_dbl) / b\_min\_a

181  / b\_min\_a / cdf\_;

182  if (!is\_constant\_struct<T\_high>::value)

183  operands\_and\_partials.d\_x3[n] -= 1.0 / b\_min\_a;

184  }

185

186  if (!is\_constant\_struct<T\_y>::value)

187  for (size\_t n = 0; n < stan::length(y); ++n)

188  operands\_and\_partials.d\_x1[n] \*= cdf;

189  if (!is\_constant\_struct<T\_low>::value)

190  for (size\_t n = 0; n < stan::length(alpha); ++n)

191  operands\_and\_partials.d\_x2[n] \*= cdf;

192  if (!is\_constant\_struct<T\_high>::value)

193  for (size\_t n = 0; n < stan::length(beta); ++n)

194  operands\_and\_partials.d\_x3[n] \*= cdf;

195

196  return operands\_and\_partials.to\_var(cdf);

197  }

198

199  template <typename T\_y, typename T\_low, typename T\_high>

200  typename return\_type<T\_y,T\_low,T\_high>::type

201  uniform\_cdf\_log(const T\_y& y, const T\_low& alpha, const T\_high& beta) {

202  static const char\* function = "stan::prob::uniform\_cdf\_log(%1%)";

203

204  using stan::math::check\_not\_nan;

205  using stan::math::check\_finite;

206  using stan::math::check\_greater;

207  using stan::math::value\_of;

208  using stan::math::check\_consistent\_sizes;

209

210  // check if any vectors are zero length

211  if (!(stan::length(y)

212  && stan::length(alpha)

213  && stan::length(beta)))

214  return 0.0;

215

216  // set up return value accumulator

217  double cdf\_log(0.0);

218  check\_not\_nan(function, y, "Random variable", &cdf\_log);

219  check\_finite(function, alpha, "Lower bound parameter", &cdf\_log);

220  check\_finite(function, beta, "Upper bound parameter", &cdf\_log);

221  check\_greater(function, beta, alpha, "Upper bound parameter", &cdf\_log);

222  check\_consistent\_sizes(function,

223  y,alpha,beta,

224  "Random variable","Lower bound parameter",

225  "Upper bound parameter",

226  &cdf\_log);

227

228  VectorView<const T\_y> y\_vec(y);

229  VectorView<const T\_low> alpha\_vec(alpha);

230  VectorView<const T\_high> beta\_vec(beta);

231  size\_t N = max\_size(y, alpha, beta);

232

233  agrad::OperandsAndPartials<T\_y,T\_low,T\_high>

234  operands\_and\_partials(y,alpha,beta);

235

236  for (size\_t n = 0; n < N; n++) {

237  const double y\_dbl = value\_of(y\_vec[n]);

238  if (y\_dbl < value\_of(alpha\_vec[n])

239  || y\_dbl > value\_of(beta\_vec[n]))

240  return stan::math::negative\_infinity();

241  if (y\_dbl == value\_of(beta\_vec[n]))

242  return operands\_and\_partials.to\_var(0.0);

243  }

244

245  for (size\_t n = 0; n < N; n++) {

246  const double y\_dbl = value\_of(y\_vec[n]);

247  const double alpha\_dbl = value\_of(alpha\_vec[n]);

248  const double beta\_dbl = value\_of(beta\_vec[n]);

249  const double b\_min\_a = beta\_dbl - alpha\_dbl;

250  const double cdf\_log\_ = (y\_dbl - alpha\_dbl) / b\_min\_a;

251

252  //cdf\_log

253  cdf\_log += log(cdf\_log\_);

254

255  //gradients

256  if (!is\_constant\_struct<T\_y>::value)

257  operands\_and\_partials.d\_x1[n] += 1.0 / b\_min\_a / cdf\_log\_;

258  if (!is\_constant\_struct<T\_low>::value)

259  operands\_and\_partials.d\_x2[n] += (y\_dbl - beta\_dbl) / b\_min\_a

260  / b\_min\_a / cdf\_log\_;

261  if (!is\_constant\_struct<T\_high>::value)

262  operands\_and\_partials.d\_x3[n] -= 1.0 / b\_min\_a;

263  }

264

265  return operands\_and\_partials.to\_var(cdf\_log);

266  }

267

268  template <typename T\_y, typename T\_low, typename T\_high>

269  typename return\_type<T\_y,T\_low,T\_high>::type

270  uniform\_ccdf\_log(const T\_y& y, const T\_low& alpha, const T\_high& beta) {

271  static const char\* function = "stan::prob::uniform\_ccdf\_log(%1%)";

272

273  using stan::math::check\_not\_nan;

274  using stan::math::check\_finite;

275  using stan::math::check\_greater;

276  using stan::math::value\_of;

277  using stan::math::check\_consistent\_sizes;

278

279  // check if any vectors are zero length

280  if (!(stan::length(y)

281  && stan::length(alpha)

282  && stan::length(beta)))

283  return 0.0;

284

285  // set up return value accumulator

286  double ccdf\_log(0.0);

287  check\_not\_nan(function, y, "Random variable", &ccdf\_log);

288  check\_finite(function, alpha, "Lower bound parameter", &ccdf\_log);

289  check\_finite(function, beta, "Upper bound parameter", &ccdf\_log);

290  check\_greater(function, beta, alpha, "Upper bound parameter", &ccdf\_log);

291  check\_consistent\_sizes(function,

292  y,alpha,beta,

293  "Random variable","Lower bound parameter",

294  "Upper bound parameter",

295  &ccdf\_log);

296

297  VectorView<const T\_y> y\_vec(y);

298  VectorView<const T\_low> alpha\_vec(alpha);

299  VectorView<const T\_high> beta\_vec(beta);

300  size\_t N = max\_size(y, alpha, beta);

301

302  for (size\_t n = 0; n < N; n++) {

303  const double y\_dbl = value\_of(y\_vec[n]);

304  if (y\_dbl < value\_of(alpha\_vec[n])

305  || y\_dbl > value\_of(beta\_vec[n]))

306  return 0.0;

307  if (y\_dbl == value\_of(beta\_vec[n]))

308  return LOG\_ZERO;

309  }

310

311  agrad::OperandsAndPartials<T\_y,T\_low,T\_high>

312  operands\_and\_partials(y,alpha,beta);

313  for (size\_t n = 0; n < N; n++) {

314  const double y\_dbl = value\_of(y\_vec[n]);

315  const double alpha\_dbl = value\_of(alpha\_vec[n]);

316  const double beta\_dbl = value\_of(beta\_vec[n]);

317  const double b\_min\_a = beta\_dbl - alpha\_dbl;

318  const double ccdf\_log\_ = 1.0 - (y\_dbl - alpha\_dbl) / b\_min\_a;

319

320  //ccdf\_log

321  ccdf\_log += log(ccdf\_log\_);

322

323  //gradients

324  if (!is\_constant\_struct<T\_y>::value)

325  operands\_and\_partials.d\_x1[n] -= 1.0 / b\_min\_a / ccdf\_log\_;

326  if (!is\_constant\_struct<T\_low>::value)

327  operands\_and\_partials.d\_x2[n] -= (y\_dbl - beta\_dbl) / b\_min\_a

328  / b\_min\_a / ccdf\_log\_;

329  if (!is\_constant\_struct<T\_high>::value)

330  operands\_and\_partials.d\_x3[n] += (y\_dbl - alpha\_dbl) / b\_min\_a

331  / b\_min\_a / ccdf\_log\_;

332  }

333

334  return operands\_and\_partials.to\_var(ccdf\_log);

335  }

336

337  template <class RNG>

338  inline double

339  uniform\_rng(const double alpha,

340  const double beta,

341  RNG& rng) {

342  using boost::variate\_generator;

343  using boost::random::uniform\_real\_distribution;

344

345  static const char\* function = "stan::prob::uniform\_rng(%1%)";

346

347  using stan::math::check\_finite;

348  using stan::math::check\_greater;

349

350  check\_finite(function, alpha, "Lower bound parameter", (double\*)0);

351  check\_finite(function, beta, "Upper bound parameter", (double\*)0);

352  check\_greater(function, beta, alpha, "Upper bound parameter", (double\*)0);

353

354  variate\_generator<RNG&, uniform\_real\_distribution<> >

355  uniform\_rng(rng, uniform\_real\_distribution<>(alpha, beta));

356  return uniform\_rng();

357  }

358  }

359 }

360 #endif

stan::prob::uniform\_log

return\_type< T\_y, T\_low, T\_high >::type uniform\_log(const T\_y &y, const T\_low &alpha, const T\_high &beta)

The log of a uniform density for the given y, lower, and upper bound.

**Definition:** uniform.hpp:44

stan::math::check\_greater

bool check\_greater(const char \*function, const T\_y &y, const T\_low &low, const char \*name, T\_result \*result)

**Definition:** check\_greater.hpp:58

traits.hpp

stan::agrad::OperandsAndPartials::to\_var

T\_return\_type to\_var(double logp)

**Definition:** partials\_vari.hpp:138

error\_handling.hpp

stan::length

size\_t length(const T &)

**Definition:** traits.hpp:159

stan::DoubleVectorView

DoubleVectorView allocates double values to be used as intermediate values.

**Definition:** traits.hpp:358

stan::math::check\_finite

bool check\_finite(const char \*function, const T\_y &y, const char \*name, T\_result \*result)

Checks if the variable y is finite.

**Definition:** check\_finite.hpp:52

partials\_vari.hpp

stan::agrad::value\_of

T value\_of(const fvar< T > &v)

Return the value of the specified variable.

**Definition:** value\_of.hpp:16

constants.hpp

stan::is\_vector

**Definition:** traits.hpp:83

stan::prob::uniform\_ccdf\_log

return\_type< T\_y, T\_low, T\_high >::type uniform\_ccdf\_log(const T\_y &y, const T\_low &alpha, const T\_high &beta)

**Definition:** uniform.hpp:270

stan::agrad::OperandsAndPartials

A variable implementation that stores operands and derivatives with respect to the variable...

**Definition:** partials\_vari.hpp:76

traits.hpp

stan::return\_type::type

boost::math::tools::promote\_args< typename scalar\_type< T1 >::type, typename scalar\_type< T2 >::type, typename scalar\_type< T3 >::type, typename scalar\_type< T4 >::type, typename scalar\_type< T5 >::type, typename scalar\_type< T6 >::type >::type type

**Definition:** traits.hpp:406

stan::is\_constant\_struct

Metaprogram to determine if a type has a base scalar type that can be assigned to type double...

**Definition:** traits.hpp:57

stan::math::value\_of

double value\_of(const T x)

Return the value of the specified scalar argument converted to a double value.

**Definition:** value\_of.hpp:24

stan::prob::include\_summand

Template metaprogram to calculate whether a summand needs to be included in a proportional (log) prob...

**Definition:** traits.hpp:35

stan::agrad::OperandsAndPartials::d\_x2

VectorView< double \*, is\_vector< T2 >::value, is\_constant\_struct< T2 >::value > d\_x2

**Definition:** partials\_vari.hpp:83

stan::prob::uniform\_rng

double uniform\_rng(const double alpha, const double beta, RNG &rng)

**Definition:** uniform.hpp:339

stan::math::check\_consistent\_sizes

bool check\_consistent\_sizes(const char \*function, const T1 &x1, const T2 &x2, const char \*name1, const char \*name2, T\_result \*result)

**Definition:** check\_consistent\_sizes.hpp:13

stan::max\_size

size\_t max\_size(const T1 &x1, const T2 &x2)

**Definition:** traits.hpp:191

stan::math::check\_not\_nan

bool check\_not\_nan(const char \*function, const T\_y &y, const char \*name, T\_result \*result)

Checks if the variable y is nan.

**Definition:** check\_not\_nan.hpp:56

stan::prob::uniform\_cdf

return\_type< T\_y, T\_low, T\_high >::type uniform\_cdf(const T\_y &y, const T\_low &alpha, const T\_high &beta)

**Definition:** uniform.hpp:125

value\_of.hpp

stan::agrad::OperandsAndPartials::d\_x1

VectorView< double \*, is\_vector< T1 >::value, is\_constant\_struct< T1 >::value > d\_x1

**Definition:** partials\_vari.hpp:82

stan::agrad::OperandsAndPartials::d\_x3

VectorView< double \*, is\_vector< T3 >::value, is\_constant\_struct< T3 >::value > d\_x3

**Definition:** partials\_vari.hpp:84

constants.hpp

stan::agrad::log

fvar< T > log(const fvar< T > &x)

**Definition:** log.hpp:15

stan::VectorView

VectorView is a template metaprogram that takes its argument and allows it to be used like a vector...

**Definition:** traits.hpp:275

stan::prob::uniform\_cdf\_log

return\_type< T\_y, T\_low, T\_high >::type uniform\_cdf\_log(const T\_y &y, const T\_low &alpha, const T\_high &beta)

**Definition:** uniform.hpp:201

stan::math::negative\_infinity

double negative\_infinity()

Return negative infinity.

**Definition:** constants.hpp:123


---

[ Stan Home Page ]

*© 2011–2014,
Stan Development Team.*
